# Evaluation of a shared decision-making intervention for pediatric patients with asthma in the emergency department

**DOI:** 10.1186/s43058-020-00010-y

**Published:** 2020-03-04

**Authors:** Kelly Reeves, Katherine O’Hare, Lindsay Shade, Thomas Ludden, Andrew McWilliams, Melinda Manning, Melanie Hogg, Stacy Reynolds, Christopher M. Shea, Elizabeth C. Burton, Melissa Calvert, Diane M. Derkowski, Hazel Tapp

**Affiliations:** 1 Department of Family Medicine Research, Atrium Health, 2001 Vail Avenue, Suite 400B Mercy Medical Plaza, Charlotte, NC 28207 USA; 2 Center for Outcomes Research and Evaluation (CORE), Atrium Health, Research Office Building, 1540 Garden Terrace, Charlotte, NC 28203 USA; 3 Department of Emergency Medicine, Atrium Health, 1000 Blythe Blvd., 306 Medical Education Building, Charlotte, NC 28203 USA; 4 Department of Health Policy and Management, UNC Gillings School of Global Public Health, The University of North Carolina at Chapel Hill, Chapel Hill, USA; 5 Community Care Partners, 1423 E. Franklin St., Suite A, Monroe, NC 28112 USA; 6 Atrium Health, 1025 Morehead Medical Drive, Suite 600, Charlotte, NC 28204 USA

**Keywords:** Implementation, Shared decision making, Innovation, Information technology, Pediatric asthma

## Abstract

**Background:**

Asthma is a difficult-to-manage chronic disease marked with associated outcome disparities including an increase rate of emergency department (ED) visits for uncontrolled asthma among patients who are most at-risk. Shared decision making (SDM) is a process by which the patient and provider jointly make a healthcare choice. SDM improves patient outcomes; however, implementation barriers of time constraints and staff availability are limitations. The use of health IT solutions may increase the adoption of SDM, but best practices for implementation are not well understood. The Consolidated Framework for Implementation Research (CFIR) is a flexible comprehensive model used to identify barriers and facilitators influencing implementation. The goal of this study is to implement an innovative web-based pediatric SDM tool in the real-world setting of two large healthcare system EDs through the following aims: (1) convene a patient, research, and ED stakeholder advisory board to oversee review of protocol and study materials prior to implementation, (2) implement the SDM intervention where providers and staff will be trained to incorporate use of this SDM intervention, (3) conduct on-going evaluation of barriers, facilitators, and implementation outcomes to tailor implementation in the EDs, (4) evaluate patient-centered outcomes of primary care utilization and changes in ED visits and hospitalizations before and after the SDM intervention, and (5) understand and document best practices for ED implementation.

**Methods:**

The CFIR model will guide the implementation evaluation. Researchers will administer surveys to the clinical team and patients at baseline, 3, 6, and 12 months to inform implementation design, determine barriers and facilitators, and resource-needs to allow for real-time process adjustments within the EDs. Focus group or key-informant interviews and analysis will provide additional feedback to the stakeholder team to iterate the implementation process. Researchers will track patient-centered outcomes including increased primary care, ED, and inpatient utilization over the duration of the study.

**Discussion:**

To advance asthma care and the field of implementation science, further research is needed to assess best practices for incorporating SDM into high-need healthcare settings such as the ED. This knowledge will facilitate improved outcomes and appropriate policy changes towards further use of SDM interventions in local and national acute care settings.

Contributions to the literature

- Asthma is a chronic lung disease that affects more than 6.2 million children. This disease can be managed but if not well controlled, it can lead to hospitalization and even death. Studies have shown shared decision making (SDM) can help patients manage their asthma, but SDM is not always part of care.
- Research is needed to learn best practices for incorporating SDM into high-need healthcare settings such as the emergency department (ED).
- This study will implement SDM in real-world settings of two large, healthcare system pediatric EDs to evaluate the implementation process with a view of understanding best practices for ED SDM implementation**.**

## Background

Asthma is an inflammatory lung disease that affects people of all ages and has significant morbidity and mortality. In the United States (US), asthma affects over 26 million people and has experienced a concerning increase in overall prevalence [1, 2]. Inner cities are epicenters for asthma health disparities in the US, with minority children 10–17 years old bearing a disproportionate share of the burden [3, 4]. Among the most visible of these disparities is the increasing rate of visits to the emergency department (ED) for uncontrolled asthma involving the most at-risk patients who may be underinsured or without a source of primary care [5–7]. Children discharged home from the ED are at much higher future risk for exacerbation than their peers [8, 9]. Risk decreases by half if pediatric patients have an appropriate treatment plan following discharge from the ED [10]. Asthma guidelines recommend a 1- to 4-week follow-up visit with a primary care provider to develop an asthma action plan after ED discharge; however, linkage to care is often delayed or lacking [9, 11]. Thus, the burden of asthma remains high with 2 million ED visits, 439,000 hospitalizations, and 3600 deaths every year [5, 12–16].

Shared decision making (SDM) is an approach where patients and providers come together to determine the best plan of care based on evidence and patient preferences [17]. Previous studies show that SDM in primary care is associated with improved outcomes for pediatric patients with asthma [12, 17–20]. Implementation and dissemination have been highlighted as a key national priority by the Patient-Centered Outcomes Research Institute (PCORI), the Agency for Healthcare Research and Quality (AHRQ), and the Institute of Medicine (IOM) [21–23]. Yet, clinical uptake of SDM has been slow in part because of the gap in understanding how best to implement and disseminate these types of complex interventions [24–26].

From earlier work, we demonstrated that a facilitated approach to implementation of SDM was associated with improved perceptions of SDM and improved ED utilization for pediatric patients [17, 18, 27–29]. Identified implementation barriers included time constraints of the clinical team in a volume-based reimbursement structure, staff turnover, and lack of availability of staff to train as health coaches. Given the resource intensiveness of this approach combined with rapidly advancing availability of new technologies, it led us to develop a health information technology (IT) solution that included the health coach role, thus removing the need for clinical staff to aid as the health coach. Specifically, several key elements of SDM are now integrated into an interactive, virtual application called the Coach McLungs^SM^ (formally known as Carolinas Asthma Coach) available on a computer or tablet. Coach McLungs^SM^ virtually incorporates the elements of in-person SDM by using a conversational style through the animated Coach McLungs^SM^ to (1) elicit patient information (symptoms, asthma severity or control level, medication adherence, triggers, and goals), (2) provide tailored education (asthma background basics, proper inhaler technique, trigger avoidance), and (3) incorporate motivational interviewing [30]. Designed to be completed prior to an asthma-specific provider visit, the application offers the potential for patients and caregivers to be better informed and have more meaningful, efficient visits with their providers. The use of this technology has the likelihood of extending the healthcare professionals’ ability to deliver personalized care with a virtual health coach.

Despite SDM in the ED being outlined as a research priority by ED physicians, there are currently no reported uses of SDM for asthma treatment in the ED [31, 32]. To assess feasibility and address potential problems with using a virtual health coach in a high-need clinical setting, we piloted Coach McLungs^SM^ in the ED. Yet to advance asthma care and the field of implementation science, further research is needed to assess best practices for incorporating SDM into high-need healthcare settings such as the ED.

### Study objectives

The goal of this study is to implement SDM in the real-world setting of two large, healthcare system EDs and evaluate the implementation process with a view to understanding best practices for ED SDM implementation. Ultimately, this knowledge will facilitate dissemination of SDM interventions into acute care settings, both locally and nationally. To achieve this goal, we will carry out the following aims: (1) convene a patient, research, and ED stakeholder advisory board to oversee review of protocol and study materials prior to implementation; (2) implement the SDM Coach McLungs^SM^ intervention at two large healthcare system EDs where providers and staff will be trained to incorporate use of this SDM intervention; (3) conduct on-going evaluation of barriers, facilitators, and implementation outcomes to tailor the implementation for use in the EDs using the Consolidated Framework for Implementation Research (CFIR); (4) evaluate patient-centered outcomes, such as increased utilization of primary care and changes in ED visits and hospitalizations before and after the SDM intervention; and (5) understand and document best practices for ED implementation to facilitate broader future implementation and dissemination in both local and national acute care settings and health service research communities.

## Methods/design

### Setting

Implementation will take place in two hospital-based ED sites. The first site is the second largest vertically integrated healthcare system in the nation located in Charlotte, North Carolina with over 12 million patient contacts per year and provides over 85% of all uncompensated care for the community and patients with Medicare and Medicaid insurance plans. Currently, the health system cares for 61,095 asthma patients, 18,281 of which are less than 18 years of age.

A second implementation site is located in Georgia. The third largest hospital in the state with 382 beds, uniquely situated in South Cobb County serving a diverse population with average demographics of 32.8% African-American and 11% Hispanic. This implementation site is the third busiest ED in the state of Georgia with over 100,000 visits annually. As a disproportionate share hospital, this site serves a significant number of patients who are uninsured or receive Medicaid benefits. Of the pediatric patients who visit the ED, approximately 70% of them receive Medicaid health care benefits.

### Characteristics of participants

At the first implementation site, over 1200 patients per year are seen in the pediatric ED for the treatment of asthma exacerbations. Fifty eight percent of those are African-American patients. Implementation will include pediatric asthma patients age 5–17 and their caregivers (most likely parents) seen for a mild to moderate asthma exacerbation in the children’s hospital ED. We anticipate reaching about 600 pediatric patients per year who are visiting the ED for acute asthma exacerbations with the intervention. Often these patients have difficulty understanding the nature of asthma as a chronic disease and adhering to treatment plans. Many patients with asthma may not have a regular source of primary care or they have multiple barriers to regularly attending primary care. (Table 1). Our second implementation site has over 800 patients per year seen in the pediatric ED age 5–17 (characteristics of asthma patients unavailable).

**Table 1:** Characteristics of asthma patients seen in the emergency department between October 2017 and September 2018

	No. of unique patients with ED visits at implementation site 1	No. of unique patients with a visit at any ED in the healthcare system
Age 5–17	1245	4436
Male	783	2530
Female	462	1906
African American	725	2073
Caucasian	242	1597
Other/unknown	278	766
Hispanic Latino	213	555
Non-Hispanic Latino	1032	3881
Medicaid/not insured	934	2710
Other payor status	311	1726

### Theoretical model

**Fig. 1:**
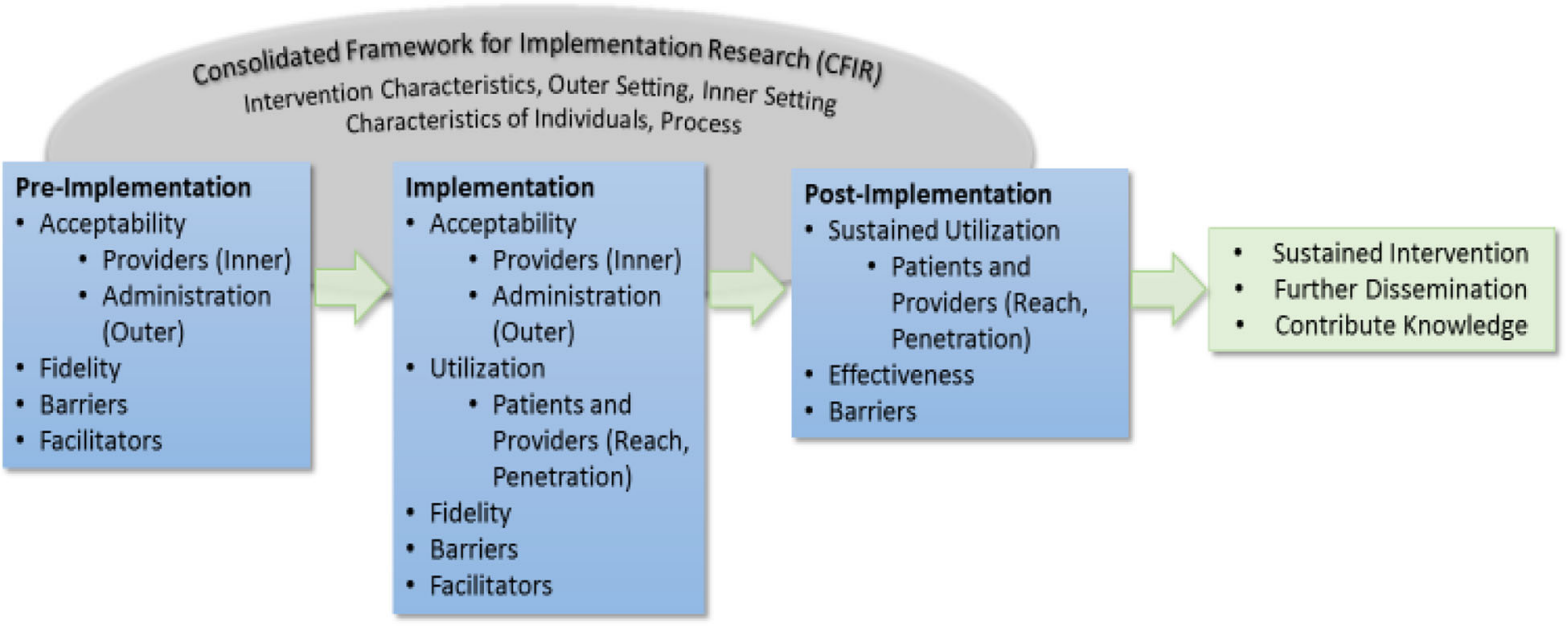
CFIR theoretical model

We will use the Consolidated Framework for Implementation Research (CFIR) to guide the implementation evaluation and inform study design throughout the research process (pre-implementation, data collection, and analysis) [33–39]. This framework represents a flexible, comprehensive model which will be used to guide and evaluate the SDM implementation. Adapting CFIR elements, such as support in the inner settings (organizational structure, culture, communication, and motivation for change) and outer settings (patient needs, resources, external policies), strength of evidence, and trialability, will be used to evaluate the intervention at all three phases of the implementation process: pre-implementation, implementation, and post-implementation [37, 40, 41] (Fig. 1).

Prior to implementing the SDM intervention, the research team will convene with an experienced group of researchers, patient advocates, providers, and implementation experts to work together as a stakeholder advisory board (SAB) (Table 2).

**Table 2:** Stakeholder advisory board members

Stakeholder advisory board	
Principle Investigator	Health Literacy Consultant
Co-Investigator	Implementation Expert, State Partner
Patient Partner	Co-Investigator
Patient Partner	Pharm D
ED Research Manager	National Advocacy Organization Vice President Corporate Affairs and Research
Child Life	Family Medicine Physician
State Partner	Physician, Asthma and QI expert
PharmD Project Lead	ED Division Chief and physician
Data Management	Patient Partner
National Expert in Asthma Research National Partner	Community Outreach Expert
National Advocacy Organization	Peds and Adult Hospitalist
Pediatrics Medical Director	Physician Family Medicine
Director of Commercialization	Pediatrician
Senior Medical Director, Pediatric Primary Care	

We will use feedback from SAB members for the iterative development of implementation evaluation tools to ensure the proposed SDM intervention is refined based on best practices appropriate for the acute care setting of the pediatric EDs.

The first step in developing measures to evaluate this SDM intervention will be to create a survey tool capable of eliciting patient and provider perspectives relative to the implementation process. Three principles will guide development: (1) ensure evaluation questions are based on the CFIR framework to include reliable measures of contextual factors known to influence implementation, (2) consider pre- and post-implementation evaluation questions to assess modifiable factors throughout the lifecycle of the study, and (3) focus on keeping the questions brief and relative to evaluating the implementation process and not the SDM intervention.

The second step of development will invite SAB members to participate in voting sessions to collect structured feedback on the efficacy of evaluation questions to identify implementation barriers and facilitators. The first round of voting will narrow down a general set of priority CFIR constructs. To develop a more detailed instrument for data collection, a second round of voting will finalize the inclusion of validated questions and selected CFIR constructs. Stakeholder feedback will allow the research team to target relevant constructs in all five CFIR domains to evaluate implementation at different time points during the study.

The final step in developing a CFIR-guided implementation evaluation tool will be to ask a health literacy expert to evaluate the acceptability of a patient-facing questionnaire. Minor changes to language and terminology may be required to improve respondent comprehension.

**Fig. 2:**
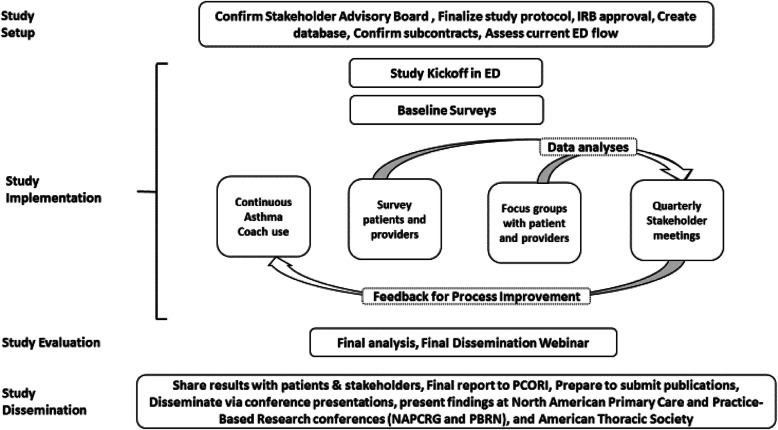
Study overview

We will use baseline assessments of implementation and ongoing evaluations to inform and adapt implementation. Mixed-methods data will be collected through surveys and semi-structured key informant interviews or focus group discussions. Feedback will be shared with the SAB to evaluate the effectiveness of implementation and process improvement cycles (Fig. 2).

### Study design

Prior to Go-Live in the ED, all providers and staff will be trained on Coach McLungs^SM^, SDM, and asthma in kick-off training sessions. To evaluate implementation, ED providers and staff will be asked to complete baseline, 3-, 6-, and 12-month CFIR surveys to evaluate the implementation process, with attention to acceptability, implementation barriers, and facilitators. A validated 3-item pediatric CollaboRate survey and 9-item questionnaire SDM-Q-DOC will evaluate the decisional process in medical encounters from the physicians’ perspectives at the specified time points [42–44]. Patient satisfaction with Coach McLungs^SM^ will be assessed using the one-item Net Promoter Score question [45–47]. Fidelity refers to ensuring the shared decision-making elements are taking place and measured through the CollaboRate and SDM-Q-DOC tools. Knowledge and self-efficacy survey questions are built into the end of the Coach McLungs^SM^ experience and adapted from items within the knowledge, attitude, and self-efficacy asthma questionnaire [48].

The number of patients with asthma diagnoses and appropriate asthma acuity for the implementation will be measured each month along with the number of patients receiving the intervention. Patient utilization data of ED visits and hospitalizations for asthma exacerbations will be extracted from the electronic data warehouse (EDW) for patients who have a diagnosis of asthma (ICD-10 code J45.XX), allowing measurement of any patient outcome improvements over the life of the study. Because of the utilization of information technology as part of the implementation through the use of the Coach McLungs^SM^, we will be able to measure how well the intervention was delivered as intended. Success will be measured through sustainability of use of the application throughout the study, improved best practice knowledge of how to implement SDM in acute care settings, and possible improved health outcomes for pediatric patients with asthma (Tables 3 and 4).

**Table 3:** Outcome Measures for Implementation Site 1

Outcomes Assessed	Assessment	Evaluation tool	Type of Outcome
Acceptability; barriers; facilitators; fidelity	Providers (Inner); Administration (Outer)	Provider CFIR Survey	Process
Acceptability	Providers (Inner)		
	Administration (External)		
Utilization	Patients (Reach)	Health Coach use vs total # eligible patients	Short Term
	Providers (Penetration)	# of Providers using tool	
Fidelity	Adherence, Exposure, Quality of Delivery	SDM_Q_DOC* (Provider)	Process
		Focus Groups	
Barriers	Characteristics of Implementation and Individual	Focus Groups	
		Provider CFIR Survey	
		Patient CFIR Survey	
Facilitators	Characteristics of Implementation and Individual	Focus Groups	
		Provider CFIR Survey	
		Patient CFIR Survey	
Acceptability	Knowledge Survey^a^	Patient Built-in - Survey	Short-Term
	Satisfaction Survey*		
	Self Efficacy Survey*		
	Decisional Conflict	CollaboRATE* (Patient)	
Effectiveness	Health Utilization Outcomes	Ed Visits	Long Term
		Hospitalizations	
		Oral Steroid Prescriptions	
		Asthma Exacerbations	
		PCP Utilization	Intermediate
Sustained Utilization	Patients (Reach)	Health Coach use vs total # eligible patients	Short Term
	Providers (Penetration)	Health Coach use vs total # eligible patients	
Barriers; Acceptability	Providers (Inner); Administration (Outer)	Quarterly Stakeholder Meetings	Process

*Surveys will include validated tools

^a^Adapted from a validated tool

**Table 4:** Outcome Measures for Implementation Site 2

Outcomes Assessed	Assessment	Evaluation tool	Type of Outcome
Acceptability; barriers; facilitators; fidelity	Providers (Inner); Administration (Outer)	Provider CFIR Survey	Process
Acceptability	Providers (Inner)		
	Administration (External)		
Utilization	Patients (Reach)	Health Coach use vs total # eligible patients	Short Term
	Providers (Penetration)	# of Providers using tool	
Barriers	Characteristics of Implementation and Individual	Provider CFIR Survey	Process
		Patient CFIR Survey	
Facilitators	Characteristics of Implementation and Individual	Provider CFIR Survey	
		Patient CFIR Survey	
Acceptability	Knowledge Survey^a^	Patient Built-in Survey	Short-Term
	Satisfaction Survey*		
	Self Efficacy Survey*		
	Decisional Conflict	CollaboRATE* (Patient)	
Sustained Utilization	Patients (Reach)	Health Coach use vs total # eligible patients	
	Providers (Penetration)		

*Surveys will include validated tools

^a^Adapted from a validated tool

### Analysis

Short, intermediate, and longer-term outcomes and evaluation plan: Results from CFIR evaluation surveys at baseline, 3, 6, and 12 months will use patient/caregiver, provider, and ED staff feedback to inform implementation design, determine barriers and facilitators, and resource-needs to allow for process adjustments. Likert scales in line with CFIR constructs will be used to collect quantitative data on selected domains and constructs including intervention characteristics (complexity, relative advantage), outer setting (patient needs and resources), inner setting (available resources, implementation climate, leadership engagement, compatibility, learning climate), characteristics of individuals (personal attributes, knowledge, and beliefs), and process (engaging). Mean ranks and *P* values will be calculated using the 5-point Likert scales. Informant interview data will be used for qualitative content analysis, with a CFIR-directed approach for coding. The categorizing process and coding will continue until saturation is reached by independent coders.

## Discussion

The goal of this study is the evaluation of a SDM intervention for pediatric patients with asthma in the ED. The hectic, and often rapid, pace of the ED makes it challenging to implement new practices and innovations to improve care around asthma management, such as SDM. Our research team and the EDs implementation team have strong records of collaboration and stakeholder engagement that will prove valuable for identifying and addressing barriers to implementation, dissemination, and incorporation of results into practice.

Possible limitations of this study were identified in our pilot. One example of a potential implementation barrier is meeting appropriate provider training needs. During the pilot, feedback was received that a few physicians did not completely understand how to use the SDM Coach McLungs^SM^ summary print out and there was variability in how physicians handled the intervention. In response to this barrier, we will incorporate this SDM training into part of standard asthma education training. We will further mitigate this through the full engagement of ED providers at the project kick-off meeting.

Timing and flow of delivering the intervention in the ED environment are potential limitations. During the pilot, we were able to use the Coach McLungs^SM^ intervention during the time patients spent in the ED undergoing albuterol nebulizer treatments (typically at least 20 min is available) or in the waiting room. We anticipate this flexibility to be an important strategy in increasing use of the application.

As mentioned previously, the need for training and sustaining a person to serve as an asthma health coach is eliminated with the use of the Coach McLungs^SM^ application. Coach McLungs^SM^ was designed to remove the need for training and sustaining personnel serving as a health coach. This is reflected in our proposed rollout within the pediatric ED (Table 5). The training roll-out will be adapted to meet their schedule, existing workflow/care pathways, and based on their needs identified during the planning phase.

**Table 5:** Rollout Training Program for ED providers

	Target Audience	Target Audience
	ED Providers (Fellows, Residents, & Attendings)	Care Team (Nurses, Health Techs)
Kick -Off and Program Training	Incorporate this SDM training into part of standard asthma education training to include: • Introductions • Pediatric Asthma- Gaps and Opportunity • Shared Decision Making • Coach McLungs^SM^ Introductions • Asthma Refresh • Inhaler Technique Practice • Workflow & Implementation Overview	Presentations during certain target shifts to ensure nurses understand the goals to improve asthma care through shared decision making and Coach McLungs^SM^ Discuss how to identify target population, and how to initiate giving Coach McLungs^SM^ to qualifying patients.
Go Live with Coach McLungs^SM^	Begin implementation and debrief/troubleshoot at meetings mentioned above. Discussions to tailor the implementation will take place on a 6-monthly basis at the meetings described above. Discussion will include identified barriers, facilitators and implementation outcomes.	

### Future dissemination and scalability

The well-established partnership between the research team, multiple patient-partners, and stakeholders is the foundation for identifying further stakeholders for this study. Broad categories of key stakeholders are patients living with asthma (patient partners), physicians and healthcare providers, implementation experts, participatory research experts, healthcare systems, advocacy groups such as local the local asthma coalition and national asthma foundation, policy makers, and funders such as state Medicaid networks with whom we have previously partnered. We will work with other policy groups to report results. As with our previous projects, we will support stakeholders, such as patients and providers partnering with researchers in giving national presentations, and authoring manuscripts to give input on their respective perspectives.

## Conclusion

This study will implement SDM in the real-world setting of two large, healthcare system pediatric EDs to evaluate the implementation process with a view of (1) improving outcomes for asthma patients and (2) understanding best practices for ED SDM implementation. We anticipate that a successful implementation of this health technology application in the EDs will improve patient outcomes particularly for those most in-need with frequent ED visits and without a regular source of primary care. Ultimately, this knowledge will facilitate improved outcomes and appropriate policy changes towards further use of SDM interventions in acute care settings both locally and nationally.

## Data Availability

Not applicable

## Abbreviations

AHRQ: Agency for Healthcare Research and Quality
CFIR: Consolidated Framework for Implementation Research
ED: Emergency department
IOM: Institute of Medicine
IT: Information technology
PCORI: Patient-Centered Outcomes Research Institute
SDM: Shared decision making
US: United States
